# The Growth of Photoactive Porphyrin-Based MOF Thin Films Using the Liquid-Phase Epitaxy Approach and Their Optoelectronic Properties

**DOI:** 10.3390/ma12152457

**Published:** 2019-08-01

**Authors:** Guy Olivier Ngongang Ndjawa, Mohamed R. Tchalala, Osama Shekhah, Jafar I. Khan, Ahmed E. Mansour, Justyna Czaban-Jóźwiak, Lukasz J. Weselinski, Hassan Ait Ahsaine, Aram Amassian, Mohamed Eddaoudi

**Affiliations:** 1 King Abdullah University of Science and Technology (KAUST), KAUST Solar Center (KSC), Physical Sciences and Engineering Division (PSE), Thuwal 23955-6900, Saudi Arabia; 2 Functional Materials Discovery and Development Research Group (FMD3), Advanced Membranes and Porous Materials Center (AMPMC), Physical Science and Engineering Division (PSE), King Abdullah University of Science and Technology (KAUST), Thuwal 23955-6900, Saudi Arabia; 3 KAUST Catalysis Center (KCC), Physical Science and Engineering Division (PSE), King Abdullah University of Science and Technology (KAUST), Thuwal 23955-6900, Saudi Arabia

**Keywords:** metal-organic framework, thin films, photoactive, optolectronic and liquid-phase epitaxy

## Abstract

This study reports on the optoelectronic properties of porphyrin-based metal–organic framework (MOF) thin films fabricated by a facile liquid-phase epitaxy approach. This approach affords the growth of MOF thin films that are free of morphological imperfections, more suitable for optoelectronic applications. Chemical modifications such as the porphyrin ligand metallation have been found to preserve the morphology of the grown films making this approach particularly suitable for molecular alteration of MOF thin film optoelectronic properties without compromising its mesoscale morphology significantly. Particularly, the metallation of the ligand was found to be effective to tune the MOF bandgap. These porphyrin-based MOF thin films were shown to function effectively as donor layers in solar cells based on a Fullerene-C_60_ acceptor. The ability to fabricate MOF solar cells free of a liquid-phase acceptor greatly simplifies device fabrication and enables pairing of MOFs as light absorbers with a wide range of acceptors including non-fullerene acceptors.

## 1. Introduction

Metal–organic frameworks (MOFs) are ordered synthetic porous solids with a high degree of long-range order [1,2,3,4]. They are chemically versatile, structurally and chemically stable, and have found their way in a wide range of applications including gas separation, gas storage, and catalysis [5,6,7,8]. This flexibility, unparalleled in other classes of microporous materials, originates from their rational design and versatile chemistry based on both the well-established metal coordination modes and the control of organic linker topologies through the application of reticular chemistry [1,2,3,4,5,6,7]. This approach allows a sheer number of combinations of metal ions and organic “linker” groups to be explored and in turn enables the design of pore size and shape and imparting chemical functionality so as to meet a wide range of technological application needs [7,8,9,10]. MOFs which can be designed to absorb or emit light and transport electrical charges are of particular interest for various applications [11,12,13,14]. For example, MOFs have been adapted for a vast variety of optical applications, including luminescent pH sensors [12], photo-catalysis [15,16,17], tunable light emitters [13], and photovoltaics [14]. One important characteristic of MOFs is that their self-assembly can be controlled through the appropriate choice of organic linkers and metal ions/clusters building blocks, that leads to the growth of an MOF with two- or three-dimensional structures. A diverse range of chemical building blocks are employed not only to control the way in which MOFs assemble in space but also to impart them with multifunctional characteristics including tailored optical and electronic properties [1,2,3,4,5,6,7]. The presence of inorganic clusters connected to organic linkers possessing complexing and chelating groups such as carboxylates and nitrogen-containing compounds affords the ability to absorb sunlight both in the ultraviolet region and the visible spectral regions [9,10,11]. However, the intrinsic optoelectronic properties of MOFs are poorly understood, and the development of efficient MOF-based materials as active layer components for optoelectronic devices is still in the early stages. 

Porphyrin-based ligands have been extensively used as organic ligands in the synthesis of MOFs. They are structurally similar to many chlorophylls with which they have resembling chromophores. Additionally, porphyrins are rigid and thermally stable and are known to exhibit structural rigidity and terminal pendant functionalities that can be tuned easily using organic chemistry [18,19]. These porphyrin-based materials represent an interesting platform of MOFs, which has received extra attention in the past decade. MOFs that are based on porphyrin ligands can thus be expected to possess unique properties particularly suitable for applications involving catalysis and light harvesting [19]. Additionally, because porphyrin-based chromophores are planar, they have reduced symmetry which favors anisotropic hopping of excitons. Their planar geometry thus affords the opportunity to control the direction of exciton transport with the MOF through controlling the orientation of the chromophores [14]. Furthermore, the ability of the porphyrin core to be chemically tuned via metallation with different cations enables the modulation of the properties of the synthesized MOF and provides the means to understand the influence of the chemical composition of the organic ligand on the overall MOF properties [18].

MOF-based devices require the deposition/growth of MOFs as thin films on functional substrates [20,21]. The fabrication or growth of MOF thin films can be achieved using different techniques including conventional solvothermal methods [20]. However, this conventional method leads to uncontrolled thickness and inhomogeneous film growth, resulting in thin films with ill-defined morphologies making it extremely challenging to achieve high quality MOF thin films [20]. An extremely versatile approach, namely the liquid-phase epitaxy (LPE, also known as layer-by-layer) MOF growth technique, was introduced in 2007 [21]. This approach relies on the sequential reaction of the functionalized substrate with the metal precursor or the organic linker without mixing reactants to allow a single layer to be grown at a time resulting in enhanced controlled MOF thin film growth [22,23]. The MOF thin films produced were more homogenous, highly oriented, and with well-defined thicknesses. Recently, MOF-based thin films, especially optical active thin films, became more attractive since they can be applied as an active component in functional devices [24,25,26]. This is due to the ability to tailor their properties via different routes, in contrast to the optical properties of conventional dense inorganic optical films which are hard to alter [27,28,29,30]. 

In this work, we present the growth of high-quality thin films of a porphyrin-based MOF on transparent conductive substrates relevant for energy harvesting applications. We used a symmetrical ditopic porphyrin-based ligand as the primary chromophore in our MOF synthesis. Like most other porphyrins, zinc-based MOF derivatives are characterized by sizable molar extinction coefficients in the blue and, to a lesser extent, red parts of the visible spectrum, making them suitable for optoelectronic applications targeting visible light. We have conducted this study to unveil key semiconducting and optoelectronic properties of porphyrin-based MOF thin film grown from both metallated and non-metallated MOFs, and the effect of metallation of the porphyrin ligand on the thin film properties. Our findings allow us to ascertain the particular suitability of these materials for solar cell applications. 

## 2. Results and Discussion

**Figure 1:**
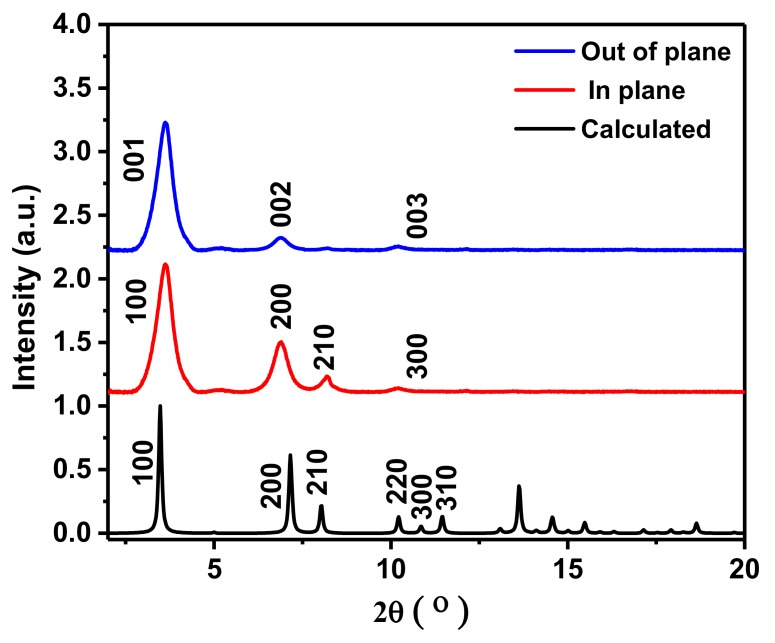
X-ray diffraction (XRD) spectra of in-plane (blue), out-of-plane (red), and simulated structure (black) of a non-metallated porphyrin-based thin film on the indium tin oxide (ITO) substrate grown using the liquid-phase epitaxy (LPE) method.

**Figure 2:**
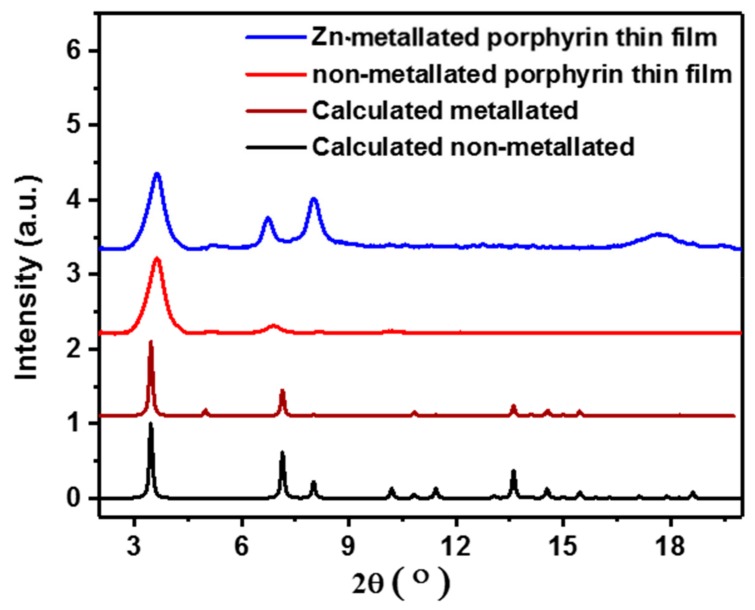
Out-of-plane XRD patterns for non-metallated (red) and Zn-metallated porphyrin-based thin films (blue) grown on the ITO substrate using the LPE method compared to the simulated structures shown in black and brown, respectively.

**Figure 3:**
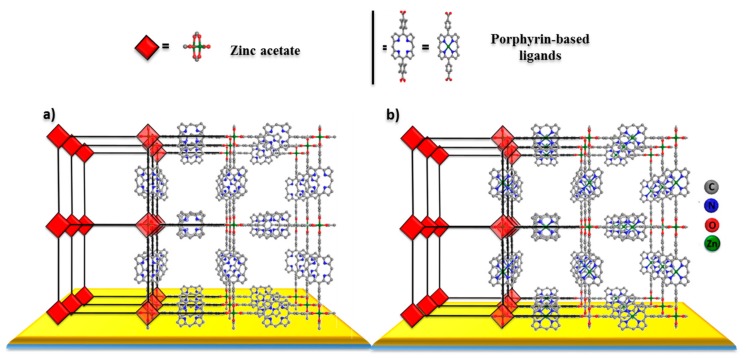
Schematic representation of the building blocks of the porphyrin-based MOF thin film. (**a**,**b**): Front view representation of the proposed structure of the highly oriented non-metallated and Zn-metallated porphyrin-based MOF thin films, respectively. Hydrogen atoms were omitted for clarity.

In order to investigate the optoelectronic and semiconducting properties of MOF thin films, we aimed first to fabricate MOF thin films with a high quality and controlled morphology using the LPE approach. We optimized the growth conditions, using the LPE approach, for a two-dimensional porphyrin-based MOF comprising the so-called paddle-wheel units [31]. The LPE approach is based on the sequential immersion of the substrate in the metal-containing precursor solution and the organic ligand precursor solution, with each growth step followed by a rinsing step in pure solvent. The main goal of this growth method is to allow the metal or ligand to react independently. The number of growth cycles then dictates the film thickness and its surface termination leading to well-controlled film growth. This approach was used to grow both non-metallated and Zn-metallated MOF films based on the 5, 15-diphenyl-10, 3, 20-di(4-carboxyphenyl) porphyrin ligand. The non-metallated and Zn-metallated porphyrin-based thin films grown with an optimized recipe (Supplementary Information) were characterized by X-ray diffraction (XRD), as shown in Figure 1 and Figure 2. Figure 1 shows the out-of-plane and in-plane XRD patterns of the non-metallated porphyrin-based MOF thin films grown on an indium tin oxide (ITO) substrate and the calculated XRD patterns of the simulated structures. The XRD patterns directly demonstrate that the LPE method yields a MOF thin film whose structure is consistent with stacked layers with an overall P4 symmetry [14]. These patterns agree well with results for similar structures from earlier studies, indicating that a pure phase of the porphyrin-based MOF was obtained [14]. The peaks appearing at 2θ of 3.4° and 6.8° are assigned to the (001) and (002) planes with respect to the calculated structure, respectively. The position of this peak allows us to determine the size of the unit cell squares; the value of 24.0 Å for the Zn-metallated MOF is in excellent agreement with that reported in previous studies [14]. The fact that no other diffraction peaks or only very weak peaks are seen reveals that these layers are highly oriented and are perpendicular to the templating substrate, as illustrated in Figure 3. The in-plane XRD pattern recorded at grazing incidence shows only one additional diffraction peak, a (210) peak, which determines the distance between the stacked layers of about 6.4 Å. The fact that the (100) and (001) diffraction peaks are located at identical positions, as seen from out- and in-plane patterns, demonstrates that the planes are regularly stacked yielding a simple tetragonal unit cell. In case there was any distortion of the unit cell, this would yield different positions of the (001) peak in the out-of-plane and of the (100) peak in the in-plane diffraction data [14,31,32]. In the case of the Zn-metallated porphyrin-based MOF thin film grown using identical conditions as for the non-metallated case, the XRD pattern also shows the formation of a highly crystalline thin film with an identical structure as can be revealed from the agreement of the XRD peaks with the simulated patterns in Figure 2. However, in this case, the films are less textured with a less preferred orientation along (001) [14].

**Figure 4:**
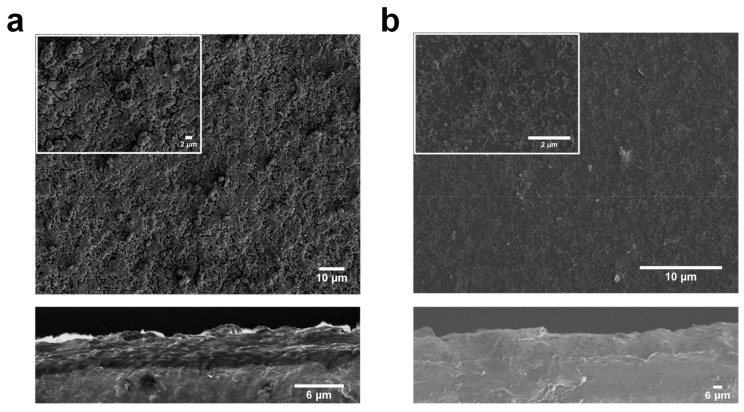
Top view and cross section scanning electron microscopy (SEM) images of the (**a**) non-metallated porphyrin thin film and (**b**) Zn-metallated porphyrin thin film grown on ITO substrates.

The fabricated porphyrin-based MOF thin films consist of a 2-D layers stack formed through self-assembly driven by the interaction of Zn-paddle-wheels and the carboxylic acid groups at the periphery of the porphyrins molecular unit (Figure 3). To investigate the surface morphology of these MOF thin films we have used scanning electron microscopy (SEM) [20,22]. In Figure 4a,b, top view and cross section SEM micrographs of the non-metallated porphyrin-based MOF thin film and the Zn-metallated porphyrin MOF thin films grown on ITO substrates are shown. The thickness was controlled by the number of deposition cycles, and in this case, these thin films were obtained after 150 cycles of growth. The cross-section images show that the film thickness is in the order of 200 nm for non-metallated porphyrin-based MOF thin films and only about 120 nm in the case of the Zn-metallated porphyrin-based MOF thin film, which we believe is due to the difference in the reactivity and interaction behavior of the metallated ligand, which affects the growth orientation. The micrographs reveal the formation of uniform, smooth, and homogeneous surfaces for both thin films, which are composed of intergrown nano-sized crystals. The nano-sized crystals are critical for the continuity, compactness, and homogeneity of the fabricated non-metallated porphyrin-based MOF thin film. More significantly, the SEM images (top view and cross section) also show the attainment of a closed thin film with no evidence of cracks. Film smoothness is critical for most applications, especially those involving light harvesting applications which are less tolerant to film imperfections [14]. The SEM images obtained at high magnification (insets in Figure 4a,b) indicate that the grains that form the polycrystalline film are relatively uniform and homogenous with the absence of any defects like pinholes or cracks. However, a few cracks are apparent as shown in Figure 4a and are mainly due to the high-energy electron beam of the SEM which is known to introduce damage in MOFs and has been reported in the literature [33,34,35]. The cross-section images of the SEM micrographs (Figure 4b) revealed a much smoother thin film morphology in the case of a Zn-metallated porphyrin-based thin film, which could be due to the slower growth of this particular thin film.

In contrast to the conventional solvothermal process, which is known to yield very rough MOF thin films, porphyrin-based MOFs grown using the LPE approach allowed for a more homogenous and controlled film growth, which is revealed in the precise control of orientation and thickness of the MOF thin film. The manner in which the MOF grows on the ITO electrodes impacts the overall film formation and determines its potential optoelectronic and energy harvesting applications. Such smooth and oriented films are more suitable for planar heterojunctions solar cells where the interfacial morphology is expected to impact photovoltaic performance critically.

**Figure 5:**
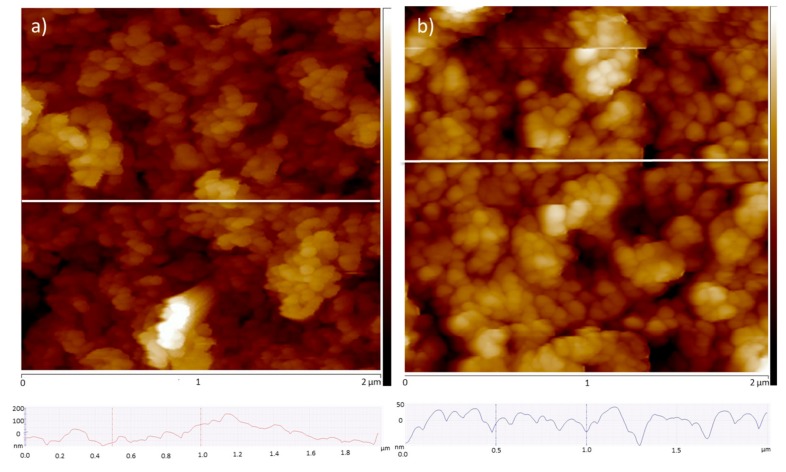
Topographic atomic force microscopy (AFM) images of (**a**) non-metallated porphyrin thin film and (**b**) Zn-metallated porphyrin thin film grown on ITO substrates, and the corresponding height-averaged profile.

In order to get more insight on the topography and surface roughness of the non-metallated and Zn-metallated porphyrin thin films grown on ITO substrates, we used the atomic force microscopy (AFM) technique. The AFM micrographs of 300-cycle samples are shown in Figure 5a,b. They reveal a rather homogeneous surfaces composed of intergrown nano-sized crystals which further confirms the effectivity of the LPE as a method for fabricating high quality thin films [21]. The roughness of the thin films was estimated using the root mean squared (RMS) of the height profiles extracted from AFM images. In the case of the non-metallated thin film the RMS roughness varied in the range 60–70 nm. For the metallated thin film RMS roughness varied in the range 40–50 nm, suggesting that these films are much smoother than those fabricated with non-metallated porphyrin and in good agreement with the SEM images. 

**Figure 6:**
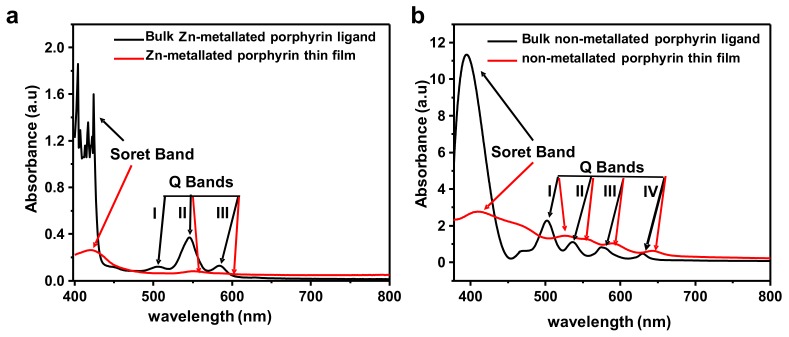
Ultraviolet-visible (UV-Vis) spectra of (**a**) bulk Zn-metallated porphyrin ligand (black) and Zn-metallated porphyrin thin film (red), and (**b**) bulk non-metallated porphyrin ligand (black) and non-metallated porphyrin thin film(red) grown on ITO substrates.

Similarly to structural and morphological properties, optical properties are equally dependent on MOF composition and thin film growth conditions. The optical properties of the samples were thoroughly investigated by ultraviolet-visible (UV-Vis) spectroscopy, and the results are show in Figure 6a,b.

The UV-Vis spectra of the non-metallated porphyrin linker consist of two distinct regions: (i) the visible and the near ultraviolet regions with a spectral range extending from 360 nm to 500 nm often referred to as the Soret band (B-band) which stems from the transition from the ground state to the second excited state (S0→S2) [14]; and (ii) the Q bands with a typical absorption range in the visible region of 400 nm–700 nm, and originates from a weak transition to the first excited state (S0→S1) at lower energies.

The formation of porphyrin-based MOF thin films onto ITO substrate had a significant impact on the absorption spectrum given the absorption of the porphyrin ligand in solution. This is primarily evidenced by a 20-nm red shift for the non-metallated porphyrin-based MOF thin film relative to the Soret band of the non-metallated porphyrin ligand, and results from an expanded π-conjunction of the porphyrins constructed into the highly oriented structure of densely packed porphyrins. The Soret band is split into two bands at 380 nm and 438 nm after the formation of the MOF thin film [14]. The Q bands in the visible region are very sensitive to metallation, which is indicated by the two Q bands at 522 nm and 554 nm as observed for Zn-metallated porphyrin thin film. The existence of two Q bands compared to the four Q bands observed for free-base porphyrin thin film clearly suggests the porphyrin macrocycle is metallated with the Zn(II) ions leading to the formation of higher D_4h_ symmetric inner nitrogen atoms in porphyrin macrocycles [14]. These results show that in case of the non-metallated porphyrin ligand, no metallation of the ligand takes place during the thin film growth. The growth of the Zn-metallated porphyrin-based MOF was only possible through starting with a Zn-metallated ligand. 

**Figure 7:**
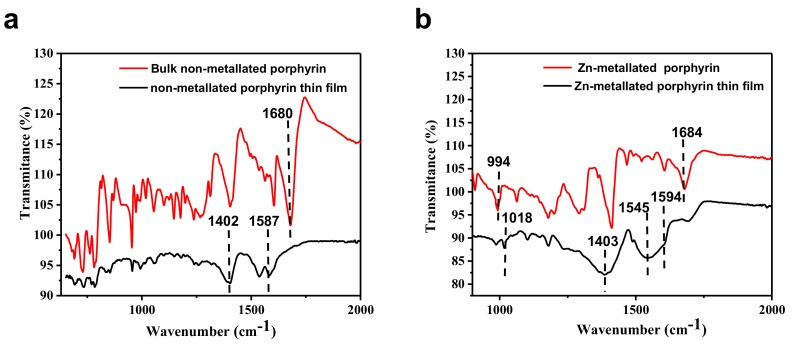
IR spectra of (**a**) bulk non-metallated porphyrin ligand (red) and non-metallated porphyrin-based MOF thin film grown on ITO and (**b**) bulk Zn-metallated porphyrin ligand and Zn-metallated 249 porphyrin-based MOF thin film grown on ITO.

Coordination between the porphyrin ligand and Zn-acetate is important for the overall MOF connectivity. To investigate the formation of these coordination bonds, the infrared spectra of both non-metallated and Zn-metallated porphyrin-based MOF thin films and corresponding non-metallated and Zn-metallated porphyrin ligands were measured (Figure 7). A strong C=O stretching band at approximately 1680 cm^−1^ is revealed in the FT-IR spectrum of the free porphyrin bulk sample. In thin films the same peak vanishes while two new peaks arise at 1587 cm^−1^ and 1402 cm^−1^, suggesting the formation of coordination bonds between the porphyrin molecules and Zn [20]. Comparative FT-IR spectra of non-metallated porphyrin-based MOF and bulk Zn-metallated-based porphyrin MOF are shown in Figure 7. It appears that after the synthesis of metalloporphyrin, no evidence of the N–H peak, typically located at around (994 cm^-1^), is observed. Since the sole difference between these two compounds is metallation of the porphyrin core with Zinc ion, the absence of a peak at 994 cm^-1^ and the appearance of one at 1018 cm^-1^ confirms the formation of the metalloporphyrin. The broad band centered at 1594 and 1403 in the FT-IR of the Zn-metallated porphyrin thin film spectrum is attributed to asymmetric and symmetric stretching of COO^-^ groups, respectively. The shift Δ = ν_asym_(COO−) − ν_sym_(COO−) can be understood as a bridging bidentate bond of carboxylate groups indicating the formation of a perfect zinc paddle-wheel structure. These results are in good agreement with a similar FT-IR analysis reported by others and which confirm the full coordination of carboxylic groups of the porphyrin ligand in the grown porphyrin-based MOFs [14].

**Figure 8:**
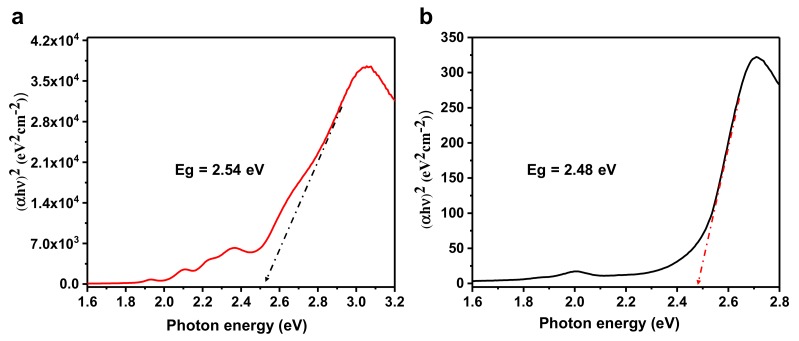
Tauc plots for two representatives, (**a**) non-metallated porphyrin-based thin film and (**b**) Zn-metallated porphyrin-based MOF thin film.

Absorbance measurements were used to determine the optical band gap of the MOFs in bulk and in thin films. Tauc plots are commonly used to extract both the band gap of a semiconductor and the nature of the band gap transition from the absorption onset. The relationship between the absorption coefficient and the energy of the photons is obtained through the following expression proposed by Tauc [36]:

(1) $${\left(\alpha h\nu \right)}^{1/n}=C\times \left(h\nu -E_{g}\right)$$

where α is the material’s absorption coefficient, ν is the frequency of light, h is Planck’s constant, C is the proportionality constant, and $E_{g}$ is the band gap energy. The exponent *n*, also known as the Tauc factor, is related to the nature of the optical transition. n  can either can take two values, either 1/2 or 2  corresponding to a direct and an indirect band gap transitions respectively. The Tauc plots for non-metallated and Zn-metallated porphyrin-based MOF thin films on the glass substrate are shown in Figure 8 and Figure S1. The band gap values were determined from the intersect of the tangent of the onset of absorption, expressed as (${\left(\alpha h\nu \right)}^{2}-{\left(\alpha h\nu \right)}^{1/2}$), with the x-axis. We found that the standard direct-gap Tauc factor (1/2) cannot provide a satisfactory fitting. However, a decent fit was obtained for n=2  in both the free base and the Zn-metallated MOF samples and suggest that our MOFs form indirect band gap semiconductors. The estimated optical gap is 2.54 eV for non-metallated porphyrin-based MOF thin film and 2.48 eV for Zn-metallated porphyrin-based MOF thin film. 

**Figure 9:**
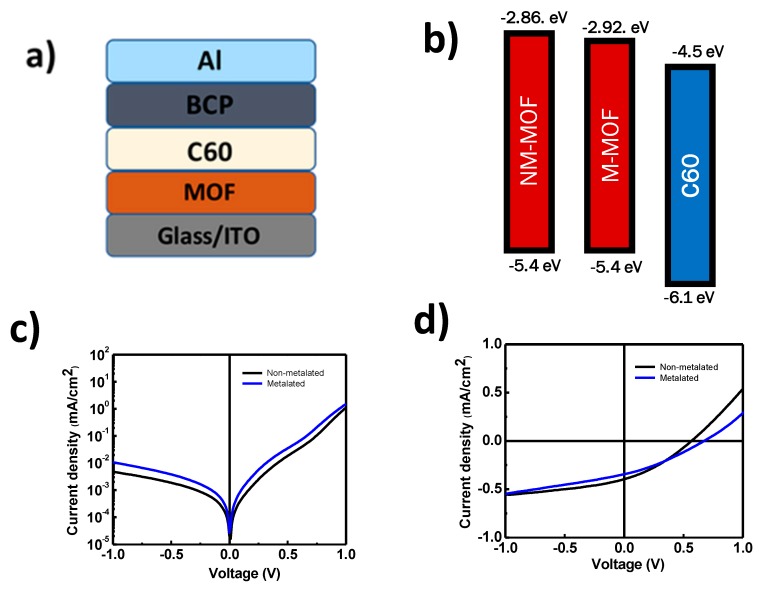
Device architecture employed for MOF solar cells (**a**) and energy levels (**b**) of the active layer materials. Current–voltage characteristics in the dark (**c**) and under illumination (**d**) for MOF/C_60_ BL solar cells.

Given the absorption properties of our porphyrin-based MOF thin films, the next step was to incorporate these films into photovoltaic cells. In this regard, growth of the MOF layer using a hole extracting layer (ITO) as a substrate is expected to lead to these films acting as absorber layers in solar cells with a bilayer architecture. To determine with which acceptor to pair the MOF, we first measure the energy levels of our MOF thin films using photoelectron yield spectroscopy in air (PYS, Figure S2) and cyclic voltammetry (Figure S3). These measurements provide the ionization energy (IE) and electron affinity (EA) of the MOF thin films. An IE of 5.4 eV was found for both non-metallated and metallated MOFs suggesting that metallation does not impact IE in this particular case. With the IEs, the EA affinity was determined from estimations based on bandgaps and further verified using cyclic voltammetry measurements. An EA of 2.86 eV was found for the non-metallated MOF while a decrease of 60 meV on this value was observed upon metalation (Figure 9b). This suggests that both non-metallated and metallated MOFs can be paired with Fullerene-C_60_ (EA of 4.5 eV), a commonly used acceptor in organic solar cells. 

The device architecture of the bilayer solar cells shown in Figure 9a consists of Glass/ITO (150 nm)/MOF (60 nm)/C_60_ (50 nm)/BCP (12 nm)/Al (100 nm) in which ITO is employed as an anode while aluminum serves as a cathode. Bathocuproine was employed as the interfacial hole blocking layer and no electron blocking was found necessary for these devices. 

Figure 9c,d show the current–voltage characteristics in the dark and under illumination of the bilayer devices and the corresponding device characteristics are summarized in Table 1.

The observed photovoltaic action in the MOF bilayer devices fabricated with C_60_ demonstrates that a heterojunction is formed between the MOF donor and the C_60_ acceptor with an energetic offset conducive for electron transfer from the MOF to C_60_. Unlike most MOF-based solar cells where liquid-phase acceptors were required to form the heterojunction, our solar cells readily form an efficient heterojunction at the donor-acceptor interface in the solid state. This is consistent with open circuit voltages (V_oc_) of both devices, which are close to or exceeding 0.6V and short circuit current (Jsc) densities in the mA/cm^2^ range. These values represent improvements on previously reported all-solid-state MOF-based solar cells in which a MOF layer was equally employed as an absorber [37]. Analysis of the rectification behavior obtained from the cell responses in the dark yield resistances >100 kilo-ohms and demonstrate low cell leakages which is consistent with SEM and AFM results and the lack of pinholes in the densely packed MOF layers. Although the overall cell efficiencies are very encouraging, device fill-factor (FF) is low (~30%) regardless of which MOF was used, suggesting that with further device engineering these compounds would yield significantly more efficient all-solid-state devices.

**Figure 10:**
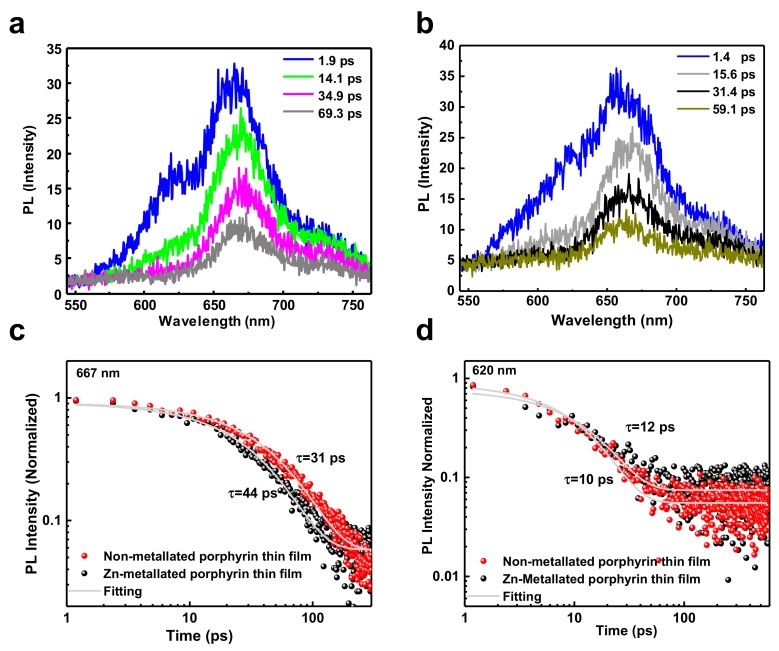
Time-resolved photoluminescence (TR-PL) data for the representative samples following optical excitation at 550 nm. (**a**) Non-metallated porphyrin thin film and (**b**) Zn-metallated porphyrin thin film display the obtained spectral evolution of the integrated signal as a function of time whilst (**c**,**d**) exhibit the transients obtained at the peak position of 667 nm and 620 nm, respectively.

Both metallated and non-metallated MOF layers exhibit the same IE suggesting they would exhibit comparable Voc when paired with the same acceptor. The oberved difference in Voc (590 mV for the non-metallated case and 670 mV for the mettalated case) suggests differences in the dynamics of the photo-excited states in donor layer. To understand the dynamics of photo-excited states in porphyrin MOFs thin films, we investigate the radiative channel decay using time-resolved photoluminescence (TR-PL). In Figure 10a,b we show the spectra of non-metallated and metallated porphyrin thin films. For both samples, the spectral feature consists of two peaks at 620 nm and 667 nm. The relative spectral decay of the non-metallated porphyrin, for which the PL signal still persists after 70 ps, is slower compared to that of the metallated porphyrin one, whose PL almost vanishes after 14 ps. In addition, the peak at 620 nm has a more rapid decay compared to the peak at 667 nm. In order to get better insight into the respective lifetimes of the excited states, the associated transients tracked at the respective peak positions are plotted and fitted with a single exponential function as shown in Figure 10c,d. As observed within a time window of 600 ps the non-metallated sample exhibits a slower dynamic and the parameterized lifetime is found to be 44 ps whilst the metallated MOF film has a lifetime of 31 ps. From the time range of 20 ps to 140 ps the decay is relatively faster for the metallated sample but eventually slows down. Analogously, the kinetics are extracted at 620 nm and presented in Figure 10d. The dynamics is relatively faster in this band compared to the previous one but is identical across the two systems providing a lifetime of 10–12 ps. 

PL measurements are further complemented by transient absorption spectroscopy (TAS). TAS was performed to investigate the picosecond–nanosecond charge carrier dynamics within a time window of 8 ns. Spectra are displayed at various time delays between the pump and probe beams of both samples (Figure S4). A broad negative photo-induced absorption (PIA) band is observed covering the spectral range from 1.3-2.1 eV in both cases. However, the signal strength is many orders of magnitude higher for the case of the non-metallated sample. In addition, a second PIA band is apparent from 2.35–2.8 eV and a narrow ground state bleach peaking at 2.2 eV is visible. The first PIA band is decaying within the first 500 ps for the non-metallated sample whereas the metallated has a prominent rapid decay in orders of 15 ps. The non-metallated sample exhibits a prominent red shift in the ground state bleach while such a shift is absent in any of the PIA bands. The fast decay is attributed to the fast quench of the singlet excitons as also observed from TR-PL measurements. Our results are in line with the findings reported in [14], and similarly we assign the fast time component to singlet exciton lifetime. The free-based porphyrin has a lifetime in orders of tens of nanoseconds, so the singlet excitons quench effectively and hence it is believed that intersystem crossing, triplet state formation, is solely responsible for the device performance. As the difference in short circuit current densities is relatively small, we can conclude that triplet state formation itself is not acting as a loss channel. However, the difference observed in the open circuit voltages is likely to stem from differences in non-radiative pathways.

In conclusion, we have successfully grown porphyrin-based MOF thin films using a simple and versatile liquid-phase epitaxial approach based on a sequential growth process from solution. This method results in thin films free of morphological imperfections and suitable for various optoelectronic applications. The impact of chemical modifications such as metallation have been found to preserve the morphology of the grown films making this approach particularly suitable for modulating/functionalization MOF thin film properties without comprising morphology significantly. In particular, metallation is found to be effective to tune the MOF bandgap. High quality MOF thin films were shown to function effectively as donor layers in solar cells based on a C_60_ acceptor. The ability to fabricate MOF solar cells free of a liquid-phase acceptor greatly simplifies device fabrication. This should enable the pairing of MOFs with a wide range of acceptors including highly absorbing non-fullerene acceptors. However, MOFs are still limiting, as the fundamental lack of uninterrupted exciton and charge transport pathways limits the intrinsic photophysical processes in thin films. The fast decay of singlet excitons limits exciton diffusion length. Future development approaches should examine pathways to increase exciton transport in thin films.

## 3. Materials and Methods

### 3.1. Substrates Cleaning and Treatment

The indium tin oxide (ITO)-coated glass substrates were cleaned in acetone for about 10 min in an ultrasonic bath, and then with plasma for about 30 min before use in order to remove surface contaminants and generate a surface with hydroxyl groups. The cleaned substrates were used immediately for the growth of MOF thin films. All characterizations were performed on ITO glass substrates.

### 3.2. The Growth of Free-Base Porphyrin Zn Thin Film and Zn-Porphyrin Zn Thin Film

Porphyrin-based thin films were fabricated using an established automated dipping system. An ethanolic solution of 20 μM 5, 15-diphenyl-10, 3, 20-di(4-carboxyphenyl) porphyrin (free-base porphyrin), [37,38] was prepared (dipping time: 10 min) and an ethanolic (VWR Company , Gliwice, Poland) solution of 1 mM zinc acetate (dipping time: 10 min) were sequentially deposited onto the substrates in a layer-by-layer fashion. In between, pure ethanol was used for rinsing the unreacted species from the surface (rinsing time: 20 s). The thickness of the sample was controlled by the number of deposition cycles. For the fabrication of the metallated Zn-porphyrin films, 200 μM solution of Zn-porphyrin was used instead of the 20 μM of free-base porphyrin. All other conditions were kept the same and preparation was done at room temperature. The resulting Zn-metallated porphyrin MOF films were relatively thinner than free-base porphyrin MOF films obtained using the same number of cycles.

### 3.3. Characterization of Free-Base Porphyrin Thin Film and Zn-Porphyrin Zn Thin Film

#### 3.3.1. X-ray Diffraction (XRD) Characterization

XRD measurements were carried out on Bruker D8 Advance diffractometers (Bruker, Karlsruhe, Germany) equipped with a position sensitive detector (PSD) LynxEye® in θ-θ geometry (Bruker, Karlsruhe, Germany), a variable divergence slit, and 2.3° Soller-slit on both primary and secondary optics were used. The XRD data was acquired over a 2θ range of 2–20° and Cu Kα-radiation (λ = 0.15419 nm) was used.

#### 3.3.2. Scanning Electron Microscopy Characterization

Cross-section scanning electron microscopy (SEM) images were obtained using a Nova NanoSEM 450 SEM (FEI) (FEI, Japan) working in the secondary electrons mode at a voltage of 2 kV and a resolution of 2 nm.

#### 3.3.3. Infrared (IR) Characterization

The infrared absorption (IR) spectra of the free-base porphyrin thin film and Zn-metallated porphyrin thin film were acquired with a resolution of 2 cm^−1^ using a Thermo Nicolet iS10 FT-IR spectrometer (Thermo Scientific, Madison, USA) under ambient conditions. 

#### 3.3.4. Ultraviolet Visible Spectroscopy (UV-Vis) Characterization

The UV-Vis spectra were recorded on a high-performance UV-Vis-NIR spectrophotometer lambda 950 (Perkin Elmer, Shelton, USA) with high photometric performance in the 250–3300 nm range. All the UV-Vis spectra were recorded in transmittance mode. 

#### 3.3.5. Atomic Force Microscopy (AFM) Characterization

The AFM images were recorded using a Bruker Dimension Icon with ScanAsyst® (Veeco Instruments Inc., San Jose, USA). All the AFM images were recorded in the tapping mode. 

#### 3.3.6. Time-Resolved Photoluminescence

For TR-PL experiments samples were excited with the wavelength-tunable output of an OPO (Radiantis Inspire HF-100) (Radian, Barcelona, Spain), itself pumped by the fundamental of a Ti:sapphire fs-oscillator (Spectra Physics MaiTai eHP) at 820 nm. The repetition rate of the fs pulses was adjusted by a pulse picker (APE Pulse Select). Typical pulse energies were in the range of several nJ. The PL of the samples was collected by an optical telescope (consisting of two plano-convex lenses) and focused on the slit of a spectrograph (PI Spectra Pro SP2300) and detected with a Streak Camera (Hamamatsu C10910) (Hamamatsu Photonics, Hamamatsu, Japan) system with a temporal resolution of 1.4 ps. The data was acquired in photon counting mode using the Streak Camera software (HPDTA) measured at room temperature and exported to Origin Pro 2015 for further analysis. 

#### 3.3.7. Transient Absorption Spectroscopy

The fundamental output of a titanium:sapphire amplifier, Coherent Legend Duo, operating at 800 nm with a repetition rate of 3 kHz and energy of 4.5 mJ was split into 3 segments (2 mJ, 1 mJ, and 1.5 mJ), and of them, two were utilized to pump two optical parametric amplifiers (Light conversion TOPAS Prime). One of the TOPAS was used to generate tunable pump pulses, while the second one generated signal (1300 nm) and idler (2000 nm). The first TOPAS was set to generate 370 nm pulses to excite the species. While the probe pathway length to the sample was kept constant at approximately 5 m between the output of the TOPAS and the sample, the pump pathway length was varied between 5.12 and 2.6 m with a broadband retroreflector mounted on a mechanical delay stage (Newport linear stage IMS600CCHA controlled by a Newport XPS motion controller), thereby achieving a variable delay between pump and probe from −400 ps to 8 ns, covering in total a time window of 8 ns. We used 1300 nm (signal) of TOPAS 2 focused onto a calcium fluoride crystal, thereby generating a white-light supercontinuum from 350 to 1100 nm.

Pump and probe beams were focused on the sample with the aid of proper optics, which was kept under a dynamic vacuum of < 10^-5^ mbar. The transmitted fraction of the white light was guided to a custom-made prism spectrograph (Entwicklungsbüro Stresing) (Entwicklungsbüro Stresing, Berlin, Germany) where it was dispersed by a prism onto a 512 pixel NMOS linear image sensor (HAMAMATSU S8381-512) (Entwicklungsbüro Stresing, Berlin, Germany). The probe pulse repetition rate was 3 kHz, while the excitation pulses were mechanically chopped to 1.5 kHz (100 fs to 8 ns delays) or directly generated at 1.5 kHz frequency (1 ns to 100 μs delays), while the detector array was read out at 3 kHz. Adjacent diode readings corresponding to the transmission of the sample after excitation and in the absence of an excitation pulse were used to calculate ΔT/T. Measurements were averaged over several thousand shots to obtain a good signal-to-noise ratio. The chirp induced by the transmissive optics was corrected with a home-built Matlab code by revaluating for each wavelength the delay at which pump and probe are simultaneously arriving on the sample as the time of the signal amplitude.

#### 3.3.8. Solar Cells Fabrication and Testing

The patterned indium tin oxide (ITO)-coated glass substrates were sequentially cleaned with soap, water, acetone, and isopropanol in an ultrasonic bath for 15 min for each step followed by a 12 min UV–ozone exposure. The MOF layer were grown using the layer-by-layer approach. The C_60_ layer was deposited in an Angstrom evaporation chamber system with a base pressure of 10^−7^ mbar on top of precleaned ITO substrates at a rate of 1 Å/s (C_60_), 0.5 Å/s (Bphen), and 1 Å/s for aluminum. The current–voltage characteristics of the bilayer solar cells were recorded in the nitrogen glove box under AM1.5 simulated illumination (ABET technology) with a spectral irradiance of 100 mW cm^−2^. The lamp intensity was calibrated using a reference silicon photodiode.

#### 3.3.9. Photoelectron Yield Spectroscopy in Air (PYS)

Measurements of photoelectron yield spectroscopy in air (PYS) were performed by using a Riken Keiki AC-2 PESA Spectrometer (Riken, Tokyo, Japan) equipped with a deuterium lamp with an energy range between 3.40 eV and 6.20 eV.

## Figures and Tables

**Table 1:** Photovoltaic performance characteristics measured under 100 mW/cm2 simulated AM1.5 irradiation. J_SC_ is short-circuit current density, FF is fill factor, and PCE is power conversion efficiency. Rsh and Rs are shunt and series resistances, respectively.

MOF Layer	Voc [V]	FF [%]	J_sc_[mA/cm^2^]	PCE [%]	Rsh (Ω.cm^2^)	Rs (Ω.cm^2^)
**Non-metallated**	0.59 ± 0.03	31.0 ± 0.5	0.37 ± 0.1	0.10 ± 0.01	2.8 × 10^4^	1074.14
**Metallated**	0.67 ± 0.03	30.1 ± 0.5	0.34 ± 0.1	0.08 ± 0.01	1.5 × 10^4^	1100.14

## Supplementary Materials

Supplementary information accompanies this paper at https://doi.org/xxxxx. Figure S1. Tauc plots for two representatives (a) free base porphyrin MOF thin film and (b) Zn-metallated porphyrin MOF thin film; Figure S2. TA spectra obtained with monochromatic excitation at 370 nm for the (a) non-metallated porphyrin-based thin film and (b) Zn-metallated porphyrin-based thin film samples; Figure S3. PESA spectra of non-metallated and metallated porphyrin MOFs; Figure S4. Cyclic voltammetry of the non-metallated porphyrin-based MOF thin film and the Zn-Metallated porphyrin-based MOF thin film in CH2Cl2 containing 0.1 M TBAPF6 using Ag/AgCl as reference electrode with a scan rate of 100 mV/s at 298 K.
