# Supplementary material for: The Growth of Photoactive Porphyrin-Based MOF Thin Films Using the Liquid-Phase Epitaxy Approach and Their Optoelectronic Properties
Source: Materials (Basel). 2019 Aug 1;12(15):2457. doi: 10.3390/ma12152457 (PMC6695608; doi:10.3390/ma12152457)
Supplement: Supplementary file 1 [file materials-12-02457-s001.pdf]

materials

MDPI

Supplemental materials

The Growth of Photoactive Porphyrin-Based MOF Thin Films Using the Liquid-Phase Epitaxy Approach and their Optoelectronic Properties

Guy Olivier Ngongang Ndjawa [1,†], Mohamed R. Tchalala [2,†], Osama Shekhah [2], Jafar I. Khan [1], Ahmed E. Mansour [1], Justyna Czaban-Jóźwiak [2], Lukasz J. Weselinski [2], Hassan Ait Ahsaine [3], Aram Amassian [1,*] and Mohamed Eddaoudi [2,*]

[1] King Abdullah University of Science and Technology (KAUST), KAUST Solar Center (KSC), Physical Sciences and Engineering Division (PSE), Thuwal 23955-6900, Kingdom of Saudi Arabia.

[2] Functional Materials Discovery and Development Research Group (FMD3), Advanced Membranes and Porous Materials Center (AMPMC), Physical Science and Engineering Division (PSE), King Abdullah University of Science and Technology (KAUST), Thuwal 23955-6900, Kingdom of Saudi Arabia

[3] KAUST Catalysis Center (KCC), Physical Science and Engineering Division (PSE), King Abdullah University of Science and Technology (KAUST), Thuwal 23955-6900, Kingdom of Saudi Arabia.

* Correspondence: aram.amassian@kaust.edu.sa (A.A.); mohamed.eddaoudi@kaust.edu.sa (M.E.); Tel.: +966-12-808-2327 (M.E.)

Received: 30 June 2019; Accepted: 25 July 2019; Published: date

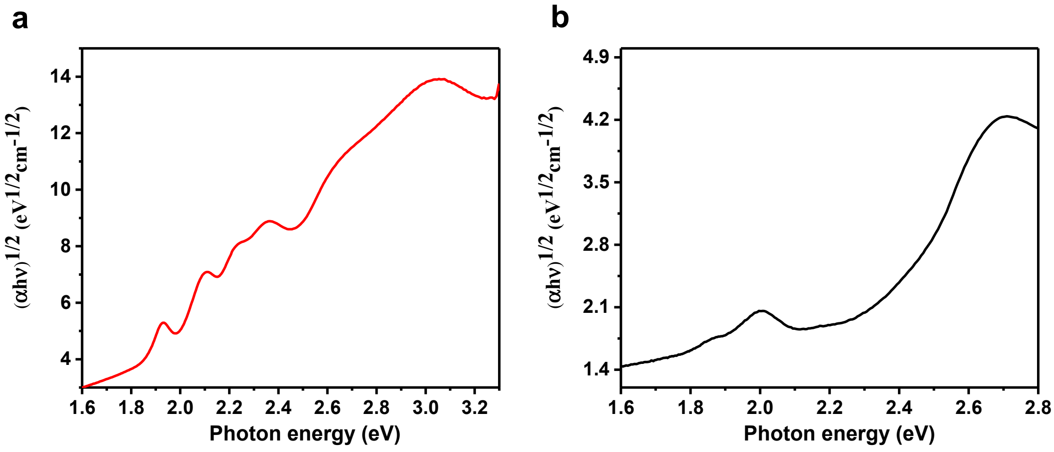

Figure S1. Tauc plots for two representatives (**a**) free base porphyrin MOF thin film and (**b**) Zn-metallated porphyrin MOF thin film.

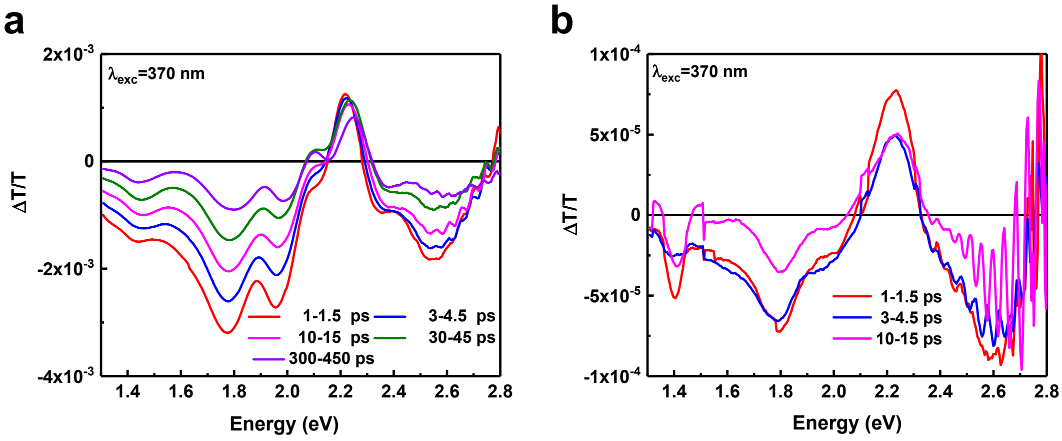

Figure S2. TA spectra obtained with monochromatic excitation at 370 nm for the (**a**) non-metallated porphyrin-based thin film and (b) Zn-metallated porphyrin-based thin film samples.

Energy levels of the MOF thin films determined by photoelectron spectroscopy in air (PESA)

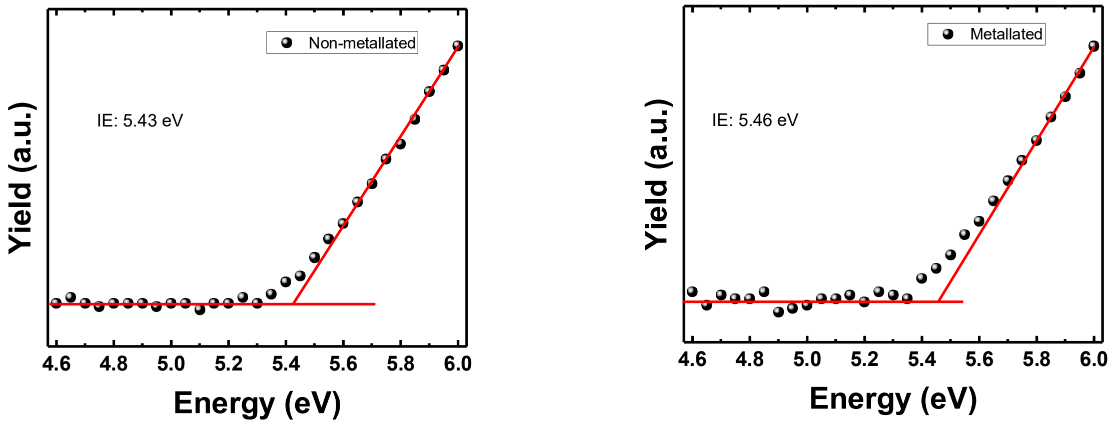

Figure S3. PESA spectra of non-metallated and metallated porphyrin MOFs.

Cyclic voltammetry

While optical properties determine the MOF's response to light, knowledge of energy level positions are key to applications involving charge separation, injection or transport. In order to elucidate the redox species in the synthesized samples, cyclic voltammetry (CV) analysis were performed under argon flow in dichloromethane CH_2Cl_2 at a 100 mV s^{-1} scan rate at room temperature. These experiments were carried out using a VMP3 Multichannel Potentiostat (BioLogic, France) at room temperature. One-compartment electrochemical cell with a three-electrode configuration was used to perform the electrochemical redox behavior. Fluorine doped tin oxide (FTO), Platinum foil and Ag/AgCl were used as working electrode, counter electrode and reference electrode, respectively.

The non-metallated porphyrin-based thin film deposited on ITO did not possess any redox behavior in CH_2Cl_2 under argon flow as shown in Figure SX. Whereas, the Zn-metallated porphyrin-based thin film sample exhibited an oxidation peak centered at 0.95 V vs Ag/AgCl and a small oxidation peak was observed at 0.59 V as shown in Figure S4. A remarkable anodic reduction was observed between 0.2–1.0 V vs. Ag/AgCl, the first pronounced anodic reduction is located at 0.83 V and a small reduction wave at 0.45 V. Additionally, an enhancement of the current density was recorded for the Zn-metallated/FTO sample.

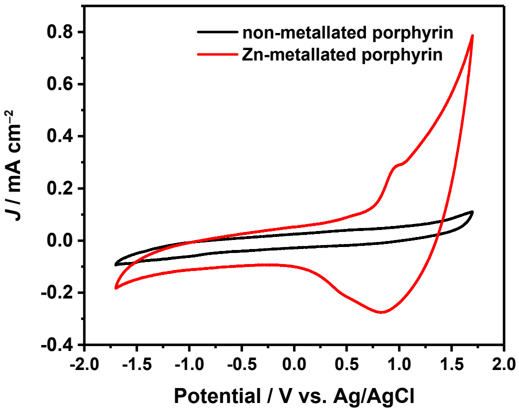

Figure S4. Cyclic voltammetry of the non-metallated porphyrin-based MOF thin film and the Zn-Metallated porphyrin-based MOF thin film in CH_2Cl_2 containing 0.1 M $TBAPF_6$ using Ag/AgCl as reference electrode with a scan rate of 100 mV/s at 298 K.
